# Heart rate variability and delirium in acute non-cardioembolic stroke: a prospective, cross-sectional, cohort study

**DOI:** 10.1007/s10072-021-05621-4

**Published:** 2021-09-29

**Authors:** Eleonora Rollo, Jessica Marotta, Antonio Callea, Valerio Brunetti, Catello Vollono, Irene Scala, Claudio Imperatori, Giovanni Frisullo, Aldobrando Broccolini, Giacomo Della Marca

**Affiliations:** 1 Department of Neurosciences, Università Cattolica del Sacro Cuore, Largo A. Gemelli, 8, 00168 Rome, Italy; 2 UOC Neuroriabilitazione Ad Alta Intensità, Fondazione Policlinico Universitario A. Gemelli IRCCS, Rome, Italy; 3 Dipartimento Scienze Dell’Invecchiamento, Neurologiche, Ortopediche e della Testa-Collo, Fondazione Policlinico Universitario A. Gemelli IRCCS, Rome, Italy; 4 Department of Human Sciences, European University of Rome, Rome, Italy

**Keywords:** Heart rate variability, Stroke, Delirium, Stroke unit, CAM-ICU, Autonomic

## Abstract

**Objectives:**

Delirium is an acute fluctuating disorder of attention and awareness. It is associated with autonomic dysfunction and increased mortality. The primary endpoint of our study was to measure autonomic activity in acute stroke patients, by means of heart rate variability analysis, in order to identify autonomic modifications that can predispose to delirium.

**Methods:**

Patients were consecutively enrolled from the stroke unit. Inclusion criteria were age ≥ 18 years and diagnosis of stroke with onset within the previous 72 h confirmed by neuroimaging. Exclusion criteria were atrial fibrillation, congestive heart failure, and conditions requiring intensive care unit. Patients were evaluated by means of Richmond Agitation Sedation Scale (RASS) and Confusion Assessment Method-Intensive Care Unit (CAM-ICU) at baseline, after 72 h, or when symptoms suggesting delirium occurred. For each patient, ECG was recorded at baseline assessment and HRV analysis was conducted on five consecutive minutes of artifact-free ECG traces.

**Results:**

Fifty-six ECGs were available for analysis. During the study period, 11 patients developed delirium. Patients with and without delirium did not differ for sex, age, severity of stroke, and comorbidities. The delirium group had greater standard deviation of the heart rate (DLR − :9.16 ± 8.28; DLR + : 14.36 ± 5.55; *p* = 0.026) and lower power spectral density of the HF component (DLR − : 38.23 ± 19.23 n.u.; DLR + : 25.75 ± 8.77 n.u.; *p* = 0.031).

**Conclusions:**

Acute non-cardioembolic stroke patients with increased variability of heart rate and decreased vagal control are at risk for delirium.

## Introduction

Delirium is an acute fluctuating disorder of attention and awareness. By definition, the diagnosis of delirium requires a decrease in attentional skills, impaired orientation in the environment, and a cognitive disturbance (e.g., memory deficit, disorientation, language, visuospatial ability, or perception) [1]. Delirium has an organic etiology, and it is not completely explained by pre-existing conditions [1]. The diagnosis of delirium is clinical, and it may be supported by several screening tools [2]. The Confusion Assessment Method for the Intensive Care Unit (CAM-ICU) is a validated diagnostic tool, widely applied in intensive care setting and recently validated for use in acute stroke patients [3, 4]. Delirium occurs in up to 30% of elderly hospitalized patients [5] and is an independent predictor of mortality during the 12 months after hospital admission [6]. In a previous study, performed in the setting of the stroke unit, we observed an incidence of delirium in acute stroke patients of 30% [7].

Several precipitating factors for delirium have been identified, or proposed [8]. It has been hypothesized that delirium may be triggered by neuroinflammation [9], imbalance in neurotransmitters [10], hypoxia [11], and aberrant response to stressors (infections, surgical traumas, anxiety [12–17]). Alterations in autonomic nervous system (ANS) activity might be involved in the pathophysiology, as well [15]. The most common tool used to indirectly evaluate autonomic function in delirium is heart rate variability (HRV). In a pilot study [15], all HRV indices at rest were higher in the delirium group, but the differences were not significant. Nevertheless, autonomic modifications associated with delirium have been investigated in few studies, in various settings, but, at the best of our knowledge, not in stroke patients.

The primary objective of this study was to evaluate heart rate variability in patients with acute stroke, in order to identify autonomic modifications that can predispose to delirium occurrence.

## Methods

### Patients

The present cohort is part of a larger study population evaluated for delirium, described in a previous paper [7].

The study design was prospective, cross-sectional. Consecutive patients were considered for enrolment in the stroke unit of the Policlinico Agostino Gemelli, Catholic University, Rome, Italy. The study went on from April to October 2020. Inclusion criteria were adult age (≥ 18 years), a diagnosis of ischemic or hemorrhagic stroke with onset of clinical symptoms within the previous 72 h, and a National Institutes of Health Stroke Scale (NIHSS) score ≥ 1 when the first clinical assessment of delirium was performed. The diagnosis had to be confirmed by neuroimaging (MRI) showing brain ischemia or hemorrhage. Exclusion criteria were transient ischemic attacks (TIA), absence of neuroimaging evidence of acute stroke (MRI), congestive heart failure, atrial fibrillation, and need for intubation and/or intensive care treatment.

All patients or caregivers gave written informed consent before enrollment. The study was performed in agreement with the Helsinki Declaration and was approved by the ethics committees of the Catholic University of Rome.

### Clinical information collected

For each patient, clinical and demographic data were collected (Table 1). At the time of first evaluation in the emergency department, neurological status was measured with the NIHSS score, and pre-existent disability was assessed by means of the modified Rankin scale (mRS) [18].

**Table 1:** Clinical and demographic data. Clinical and demographic characteristics of the study population. LVO: Large Vessel Occlusion; mRS: modified Rankin Scale; NIHSS: National Institutes of Health Stroke Scale. Statistical comparison was performed between the subgroup of patients with delirium (DLR+) and without delirium (DLR–)

	Total (*n* = 56)			Delirium − (*n* = 45)			Delirium + (*n* = 11)			Mann–Whitney	Fisher exact test
	Tot	%	Mean ± SD	Tot	%	Mean ± SD	Tot	%	Mean ± SD	*p*	*p*
Sex (male)	32	57.1		25	55.6		7	63.6			0.741
Age			69.9 ± 13.2			69.9 ± 13.2			70.0 ± 14.1	0.869	
Left side stroke	26	46.4		20	44.4		7	63.6			0.313
LVO	20	35.7		14	31.1		6	54.6			0.174
mRS			0.3 ± 0.7			0.2 ± 0.6			1.0 ± 1.1	0.101	
NIHSS			7.4 ± 6.5			6.8 ± 6.5			10.0 ± 6.2	0.106	
Impaired consciousness	4	7.1		2	4.4		2	18.2			0.169
Sedation	10	17.9		7	15.6		3	27.3			0.393
Renal failure	3	5.4		2	4.4		1	9.1			0.459
Hypertension	35	62.5		31	68.9		4	36.4			0.144
Diabetes	13	23.2		10	22.2		3	27.3			0.685
Heart diseases	18	32.1		13	28.9		5	45.5			0.268
Thyroid diseases	7	12.5		7	15.6		0	0.0			0.328
Cognitive impairment	1	1.8		0	0.0		1	9.1			0.182
Previous stroke	7	12.5		6	13.3		1	9.1			1.000

### Assessment of delirium

Delirium was diagnosed clinically with the support of two diagnostic tools: the Richmond Agitation Sedation Scale (RASS) and the Confusion Assessment Method-Intensive Care Unit (CAM-ICU) [2, 19, 20].

The RASS [20] measures the patients’ level of agitation and sedation; it includes 11 categories, numbered between − 5 and + 4, ranging from “unarousable” up to “combative” [20].

The CAM-ICU score [3, 19] is an adaptation of the Confusion Assessment Method (CAM) score for use in ICU [19, 20]. According to the CAM-ICU, a patient has delirium if:

- RASS score ≥  − 3 AND
- Acute onset change in mental status or fluctuating course in mental status, AND
-  > 2 errors in letters attention test, AND
- Either RASS is not 0, *or* combined number of errors to questions and commands is greater than 1.

The first evaluation was performed at baseline, further assessments were repeated 72 h after admission, or whenever patients displayed any of these symptoms: fluctuation in mental status, altered consciousness, fluctuating attention, or disorganized thinking.

### ECG recordings

After enrollment in the study, patients underwent bipolar chest lead electrocardiogram (ECG). Sampling rate was 512 Hz. Respiratory rate was extracted from electrocardiographic recordings for each patient. ECG registrations were performed at baseline evaluation within 24 h from admission. If a patient scored CAM-ICU positive at baseline evaluation, ECG recording was excluded from HRV analysis. Patients with non-sinus rhythm at the time of ECG recording were excluded from the analysis.

### Heart rate variability

ECG traces were extracted and converted in ASCII format for HRV analysis. ECG recordings were first analyzed visually, and epochs of recording displaying extrasystoles, body movements, or other artifacts were excluded from the analysis. Five consecutive minutes of artifact-free ECG were used for the HRV analysis. Only patients with sinus rhythm in their ECG were included in the present study. A dedicated software was used for the evaluation of the HRV parameters (HRV Analysis Software, Biomedical Signal Analysis Group, Department of Applied Physics, University of Kuopio, Finland) [21]. HRV analysis was performed both in the time domain and in the frequency domain.

#### Functional and clinical significance of HRV measures

In the time domain, the following parameters were calculated: mean HR, STD, mean RR, SDNN, RMSSD, NN50, pNN50, triangular index, TINN, SD1, and SD2.

Mean HR is 1-min heart rate. STD is the standard deviation of HR. Mean RR is the mean interval between consecutive R waves. SDNN is the standard deviation of the RR interval (N–N stands for normal-to-normal intervals; i.e., intervals between consecutive QRS complexes resulting from sinus node depolarization). RMSSD is the root mean square of consecutive differences of NN intervals; it is considered expression of vagal activity. NN50 is the number of NN intervals which differ > 50 ms from the previous interval. pNN50 is calculated by dividing NN50 by the total number of NN intervals. The HRV triangular index is the integral of the density distribution (that is, the number of all NN intervals) divided by the maximum of the density distribution. The triangular interpolation of NN interval histogram (TINN) is the baseline width of the distribution measured as a base of a triangle approximating the NN interval distribution (the minimum square difference is used to find such a triangle). SD1 describes the magnitude of the beat-to-beat variability, reflecting vagal modulation of HRV and is strongly correlated with the HF spectral component. SD2, on the other hand, describes the fluctuations in mean R–R interval over the 24-h period.

In the frequency domain, HRV was analyzed using a non-parametric autoregressive model. Two frequency bands were computed as follows: low frequency (LF; 0.04–0.15 Hz) and high frequency (HF; 0.15–0.40 Hz). Power spectral density (PSD) of LF and HF was expressed as absolute power (ms^2^), relative power (%), and in normalized units (n.u.). HF expresses parasympathetic modulation; LF reflects simultaneously sympathetic and parasympathetic control on the sinus node [22]. The ratio LF/HF, a marker of sympatho-vagal balance, was calculated.

Normalization was performed according to the formula:

$$Z=\frac{X-\mu }{\sigma }$$

where $\mu =E[X]$ is the mean and $\sigma =\sqrt{Var(X)}$ the standard deviation of the probability distribution of *X*.

A detailed description of HRV analysis, standard techniques of measurement, physiological interpretation, and clinical use is available in the report of the taskforce of the European Society of Cardiology and the North American Society of Pacing and Electrophysiology [23].

### Statistical analysis

#### Sample size

The study sample size was calculated assuming the LF and HF power spectral density as the primary endpoint variables. The G*Power [24, 25] software (version 3.1.9.6) was used for calculation. The study was planned for comparison between independent cases (patients with delirium) and controls (patients without delirium), assuming a 0.3 proportion of delirium in a series of patients admitted to stroke units [7]. The following settings were used: probability of a type I error < 0.05, effect size *d* = 0.8, power (1 − *β* error probability) = 0.8. The resulting sample size was 56 subjects.

#### Data analysis

All numerical variables were tested for normal distribution, by means of the Shapiro–Wilk test. Patients were divided into two subgroups: patients with delirium (DLR +) and without delirium (DLR −). Numerical variables are presented as mean ± standard deviation; categorical variables are presented as number (*n*) and percentage. All study variables were compared between the subgroups DLR + vs DLR − . The variables analyzed are listed in Tables 1 and 2. In order to compare numerical variables, we used a non-parametric test (Mann–Whitney *U*-test); for categorical variables, we adopted the Fisher exact test. The association of delirium and HRV parameters was evaluated in a multivariate logistic regression analysis with potential confounders as covariates (sex, age, NIHSS score, presence of large vessel occlusion (LVO), stroke side, respiratory rate, and comorbidities). The level of significance was set at *p* < 0.05.

**Table 2:** HRV analysis and comparison between DLR + and DLR − subgroups. HRV parameters in DLR+ and DLR– groups and statistical comparison. For the abbreviations see the Methods section (*2.5.1 Functional and clinical significance of HRV measures*)

	Delirium − (*n* = 45)		Delirium + (*n* = 11)		Mann–Whitney	
	Mean	SD	Mean	SD	*U*-test	*p*
Time domain						
Mean HR (1/min)	71.28	17.52	82.87	21.12	300.0	0.075
STD (1/min)	9.16	8.28	14.36	5.55	320.0	0.026
Mean RR (s)	0.90	0.18	0.78	0.18	154.0	0.540
SDNN (ms)	79.39	59.32	104.46	70.88	300.0	0.279
RMSSD (ms)	105.37	94.81	138.41	92.01	304.0	0.244
NN50 (count)	97.20	98.79	151.60	102.55	317.5	0.149
pNN50 (%)	28.31	27.48	38.39	26.23	285.0	0.439
Triangular index	0.08	0.05	0.11	0.08	285.0	0.439
TINN (ms)	458.56	342.53	680.50	367.09	338.0	0.062
SD1 (ms)	77.91	69.65	104.51	67.81	308.0	0.212
SD2 (ms)	84.99	54.99	113.87	75.18	288.0	0.404
Frequency domain						
LF (ms^2^)	1454.45	4651.00	7304.00	21,122.00	293.5	0.342
HF(ms^2^)	1890.00	4253.00	5375.00	14,201.00	289.0	0.392
LF (%)	30.01	23.57	38.71	21.01	312.5	0.179
HF (%)	46.59	23.75	53.60	16.57	278.0	0.529
LF (n.u.)	24.06	20.06	20.83	15.52	241.5	0.901
HF (n.u.)	38.23	19.23	25.75	8.77	143.0	0.031
LF/HF	0.86	0.97	0.81	0.52	287.5	0.409
Respiratory rate (breaths/min)	17.66	2.16	18.61	2.33	305.0	0.235

All statistics were performed by means of the Statistical Package for Social Science (SPSS®) software version 22 (SPSS, Inc., Chicago, IL, USA).

## Results

**Fig. 1:**
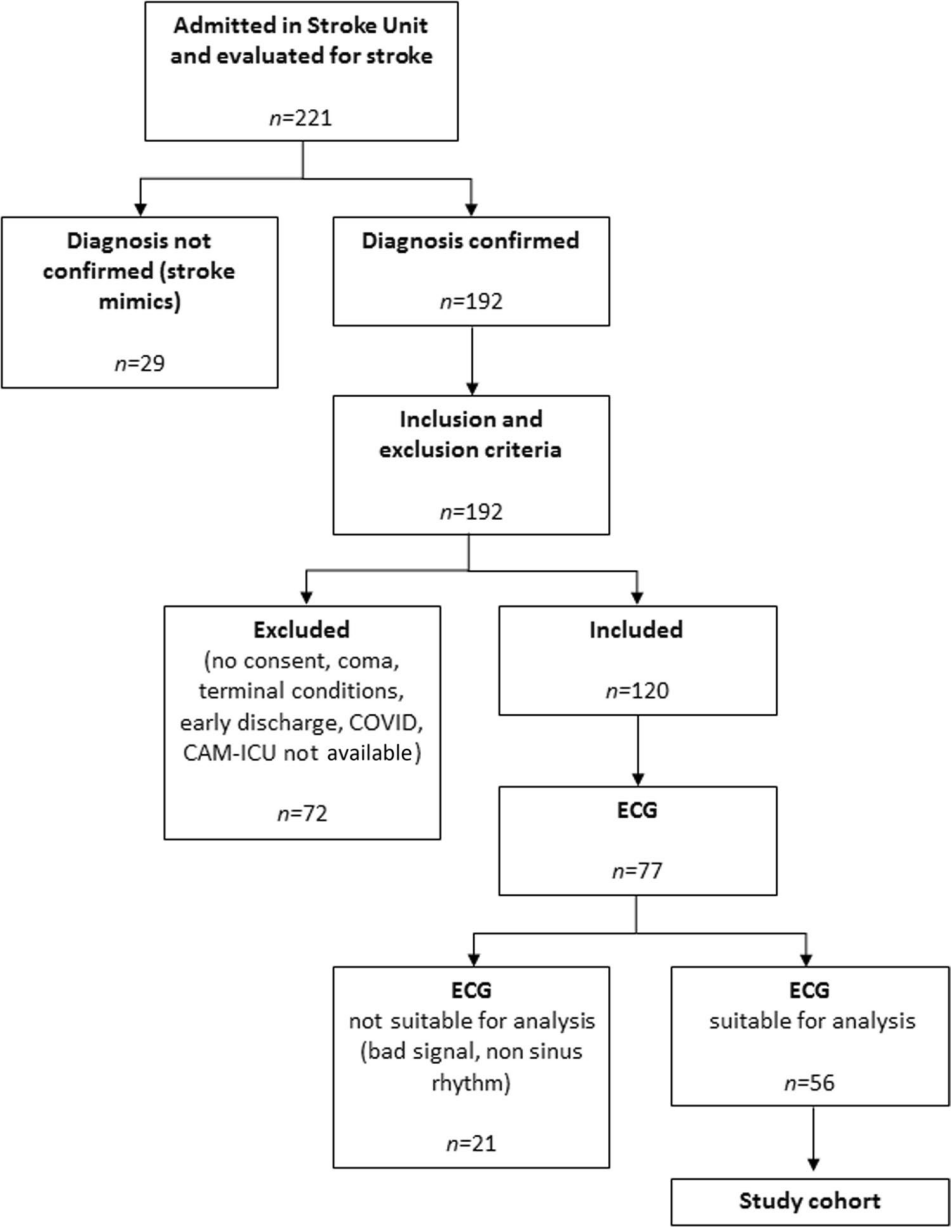
Study cohort. CONSORT diagram showing the enrolment process

During the study period, a total of 221 patients were admitted in the stroke unit and evaluated for enrolment. One hundred one patients did not meet the inclusion criteria. Useful ECG recordings were obtained in 77 patients. After excluding ECG recordings for presence of artifacts or atrial fibrillation, a total of 56 patients’ ECG traces were available for the analysis (Fig. 1). Patients with ECG recordings available for the analysis were 32 men and 24 women; mean age was 69.9 ± 13.3 years. Demographic and clinical features of the study cohort are reported in Table 1.

At the time of enrolment to study, no patient had clinical signs or CAM-ICU scores consistent with the diagnosis of delirium. The duration of the study period (that is, the permanence in stroke unit) was on average 7 days. During this period, 11 patients developed delirium, and the study cohort was divided into two subgroups: patients with delirium (DLR + , 7 men and 4 women; mean age 69.9 ± 14.1 years) and patients without delirium (DLR − , 32 men and 13 women; mean age 69.9 ± 13.2 years). The two subgroups of patients did not differ in age, sex distribution, occurrence of large vessel occlusion, stroke severity measured by means of NIHSS, stroke side, and comorbidities (Table 1). No differences in drug use upon enrolment were observed (Table 3).

**Table 3:** Drug use upon enrolment. Drugs in use at the moment of enrolment are reported for the two subgroups. Results of the Fisher exact test are also reported. ACE: angiotensin converting enzyme; AEDs: anti-epileptic drugs

	Delirium − (*n* = 45)	Delirium + (*n* = 11)	*p*
Alpha blockers	3	0	1.000
Beta blockers	9	5	0.119
Calcium channel blockers	7	1	1.000
Anti-arrhythmics	2	1	0.488
ACE inhibitors/sartans	23	6	1.000
Antipsychotics	1	0	1.000
Antidepressants	2	0	1.000
AEDs	0	0	na

### Heart rate variability analysis

In the time domain analysis, the delirium group had greater standard deviation of the heart rate (DLR − : 9.16 ± 8.28; DLR + : 14.36 ± 5.55; *U*-test: 320.0; *p* = 0.026). Also, a trend toward higher heart rate in DLR + was observed, though it did not reach statistical significance (DLR − : 71.28 ± 17.52 beats per minute; DLR + : 82.87 ± 21.12 beats per minute; *U*-test: 300.0; *p* = 0.075). No other significant difference was observed.

**Fig. 2:**
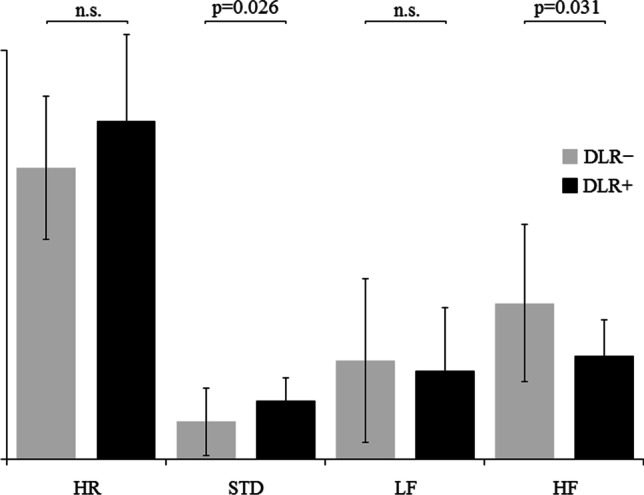
Frequency domain analysis. Comparison of HRV parameters in DLR+ (delirium) vs DLR– (no delirium) patients. HR: heart rate (beats per minute); STD: standard deviation of heart rate; LF: low-frequency component; HF: high-frequency component; n.s.: not significant

In the frequency domain analysis, patients in the delirium group had a smaller HF component, as expressed in normalized units (DLR − : 38.23 ± 19.23 n.u.; DLR + : 25.75 ± 8.77 n.u.; *U*-test: 143.0; *p* = 0.031) (Fig. 2). No other significant difference was observed. Respiratory rate did not differ between the two groups (*U*-test = 305.0; *p* = 0.235).

The association between delirium and decreased HF_nu was still significant, in a logistic multivariate regression analysis (with delirium as dependent variable), after adjustment for sex, age, NIHSS score, presence of LVO, stroke side, respiratory rate, and comorbidities.

**Fig. 3:**
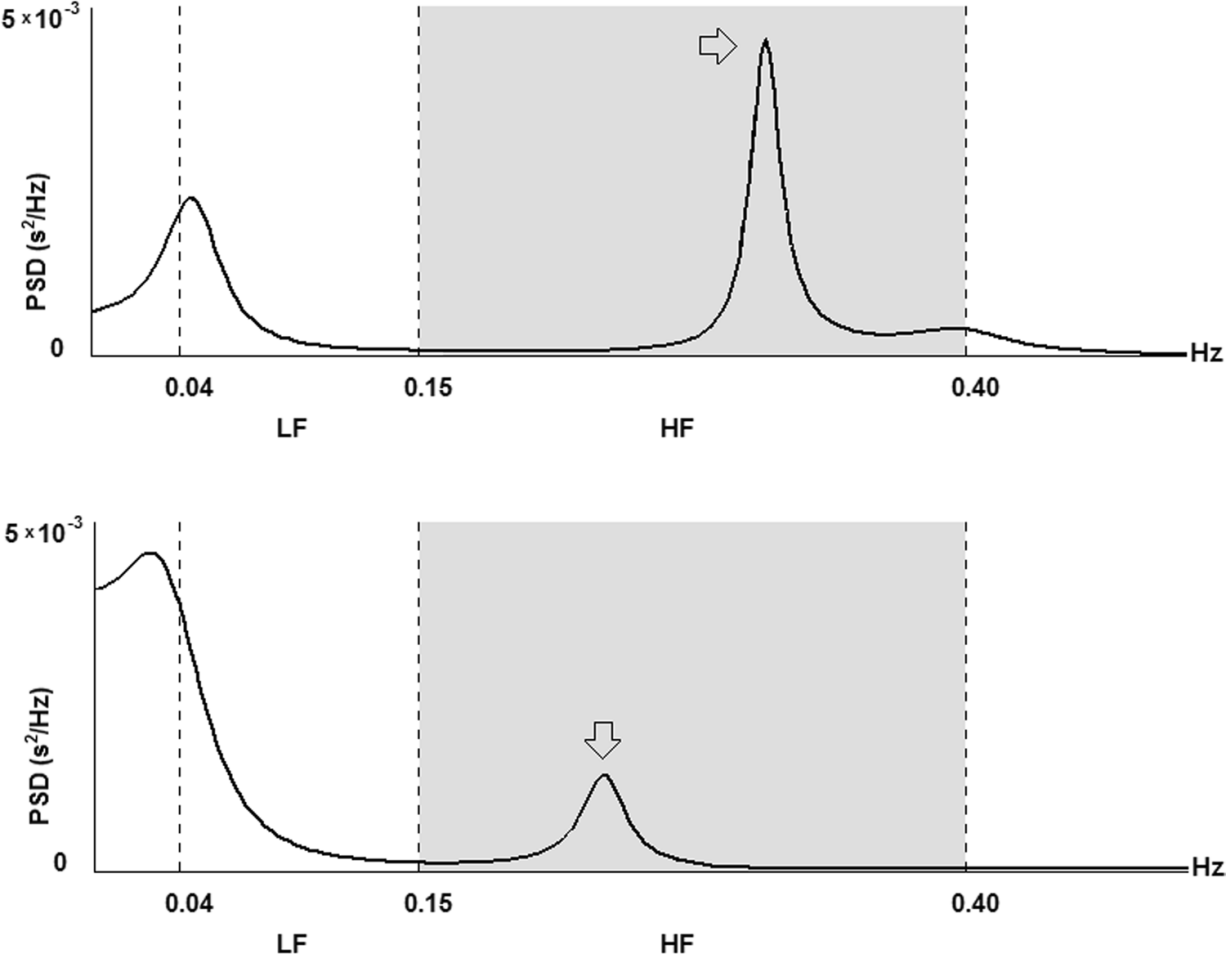
HRV frequency domain analysis in two patients from the DLR + and DLR − groups. Heart Rate Variability (HRV) analysis in frequency domain; autoregressive model. Grey panel indicates the HF portion of the power spectrum (0.15-0.40 Hz). Upper panel: a patient from the DLR– group. Lower panel: a patient from the DLR+ group. The HF spectral peak (arrows) is much smaller in the DLR+ patient than in the DLR–. LF: low frequency; HF: high frequency; PSD: power spectrum density

Detailed results of the HRV analysis and comparison between DLR + and DLR − subgroups are reported in Table 2 and in Fig. 2; examples of HRV frequency domain analysis in two patients from the DLR + and DLR − groups are shown in Fig. 3.

## Discussion

In this study, we investigated the presence of autonomic alterations, explored through HRV analysis, in acute stroke patients before the development of delirium. In our cohort, delirium occurred in patients with increased instability of heart rate (greater standard deviation of HR) and reduced vagal control, as expressed by smaller power spectral density of the HF component in frequency domain analysis. The patients who developed delirium also showed higher mean heart rate (82.9 vs 71.3 bpm), though not reaching a statistically significant difference.

These results, overall, confirm the hypothesis that delirium is associated with autonomic impairment.

Only few studies in literature have addressed this issue, and very few have performed HRV analysis. A search of the PubMed database, with the keywords “delirium” and “autonomic” or “heart rate variability,” performed in April 2021, allowed to retrieve only six papers [12, 13, 15–17, 26]. Conversely, no studies are currently available in literature exploring autonomic modifications associated with delirium in acute stroke patients.

A more variable and irregular heart rate in patients with ongoing delirium has been observed by Oh et al., in a study performed in ICU patients [13]. The same authors, in a later paper, concluded that HRV is a potential predictor of delirium [16]. However, the few studies available in literature do not show consistent results. Neerland et al. [15] observed a slightly, not significantly lower LF/HF ratio (*p* = 0.06) in patients with delirium during tilt test, suggesting a relative prevalence of vagal modulation. However, this study was conducted in a geriatric ward, not in an intensive care setting. A similar finding (decreased LF and increased HF, with lower LF/HF) was reported by Ernst et al. [12]; these authors also reported increased SDNN in patients with delirium. Zaal et al. [17], in one of the earliest studies on HRV and delirium, could not measure any difference in HRV parameters between patients with and without delirium; this result was probably due to the low numerosity of their sample [25 patients in total]. These authors excluded patients with any history of neurological diseases, because they were considered associated with expected autonomic alterations.

Even though in literature the role of autonomic control in atrial fibrillation (AF) is not clearly established, AF has been traditionally considered an exclusion criterion from HRV studies [27]. Accordingly, patients with AF were not included in our study. However, as shown in our previous paper, the prevalence of delirium does not differ between the cohorts of patients with and without AF [7].

In our study, we investigated delirium occurrence in a condition (i.e., acute stroke) which itself could be associated with impaired or aberrant autonomic activity. For instance, a predominance of sympathetic tone could be present in acute stroke to sustain blood cerebral perfusion in the presence of large vessel thrombosis. To exclude the effect of potential confounders, we compared the HRV parameters between the two groups with adjustment for the presence of LVO. Even after correction for LVO, HF_nu was still significantly smaller in the group of patients with delirium (*p* < 0.001). Moreover, after adjustment for the severity of stroke, expressed by NIHSS score, HF_nu was still significantly smaller in the DLR + group (*p* = 0.04). It is worth noticing that in our study risk factors traditionally associated with delirium occurrence, such as age and stroke severity [28–32], were not different between the two subgroups. This may reflect the specific exclusion criteria adopted, in particular the choice to exclude patients with AF. Notably, HF decrease is not associated with LF modification. In an experimental model in healthy subjects, after the administration of propranolol and methylatropine, HRV parameters decreased and virtually neared zero, both in time and frequency domain [27]. Indeed, it may happen that both LF and HF components are reduced, even if in our population the LF reduction did not reach statistical significance. As a consequence, the LF/HF ratio is substantially the same in the two groups.

A pathophysiological model of delirium has recently been proposed, where direct brain insults together with aberrant stress response concur in delirium development. In this “stress response system,” the autonomous nervous system plays a substantial role, contributing to delirium pathogenesis [33]. In particular, a depressed parasympathetic activity has been postulated as the pathophysiological basis for the development of ICU delirium [34]. This imbalance between sympathetic and vagal systems is even more pronounced when dealing with the hyperactive subtype of delirium, which has been interpreted as a hyperadrenergic autonomic dysfunction by the American College of Emergency Physicians (ACEP) Task Force on Excited Delirium Syndrome [35].

In our study, we found a relative decrease in parasympathetic activity with respect to sympathetic activity in patients who will later develop delirium. These results confirm the hypothesis suggested by Maclullich et al. [33] that delirium pathophysiological basis is partially explained by an imbalance in the autonomous nervous system. Moreover, our data allow to extend this model to acute, non-cardioembolic, stroke patients.

The functional status of the autonomic nervous system, derived from the non-invasive measurement of HRV, represents an important prognostic information regarding mortality risk in cardiovascular diseases [36–39]. Autonomic nervous system imbalance is a prognostic factor in acute stroke as well, as changes in frequency and time domain HRV parameters have been correlated with functional outcome and with mortality [40]. Traditionally, depressed HRV has been considered an adverse prognostic factor in several clinical studies [41, 42]. However, further studies have demonstrated that also abnormally increased HRV parameters, such as SDNN, are associated with higher risk of mortality [43].

On the other hand, the occurrence of delirium in acute phase of stroke negatively impacts the long-term prognosis of patients [44]. As both represent independent predictors of outcome in acute stroke, it is important to establish a possible correlation between autonomic system impairment and delirium. Our study suggests that an alteration of HRV may help to identify patients at risk for delirium development, and therefore may guide physicians to direct intervention strategies to prevent delirium [45].

This study has some limitations. First, the study was performed in a cohort of acute stroke patients with several comorbidities and receiving multiple pharmacological treatment. Nevertheless, statistical comparison between the drugs in use upon enrolment in the two groups did not show any differences. Moreover, after correction for age, sex, NIHSS, presence of LVO, side of the lesion, breathing rate, and comorbidities (including diabetes), the analysis still confirmed a significantly lower HF_nu in patients who later developed delirium.

In spite of these limitations, the strength of our study is the electrocardiographic assessment prior to delirium development: the alterations in the HRV parameters do not represent the consequence of the autonomic activation which takes place in ongoing delirium state, but are rather an anticipating event of delirium. In addition, our study was conducted on a homogenous cohort of patients, which did not include conditions that could represent potential confounders, such as TIA, coma, and dementia. Indeed, we enrolled only one patient with mild cognitive impairment and none with dementia.

## Conclusion

This is the first study exploring heart rate variability as predictor of delirium in acute stroke patients. Increased instability of heart rate and decreased vagal output are associated with development of delirium. The early detection of such pathophysiological parameters may help reduce delirium occurrence, as it may prompt intervention strategies to prevent delirium.

## Data Availability

Data are available on request.

## References

[1] Association AP (2013) Diagnostic and statistical manual of mental disorders (DSM-5®). American Psychiatric Pub 10.1590/s2317-17822013000200017 24413388

[2] De J, Wand APF (2015). Delirium screening: a systematic review of delirium screening tools in hospitalized patients. Gerontologist.

[3] Mitasova A, Kostalova M, Bednarik J, Michalcakova R, Kasparek T, Balabanova P (2012). Poststroke delirium incidence and outcomes: validation of the confusion assessment method for the intensive care unit (CAM-ICU). Crit Care Med.

[4] Fleischmann R, Warwas S, Andrasch T, Kunz R, Witt C, Mengel A (2021). Course and recognition of poststroke delirium: a prospective noninferiority trial of delirium screening tools. Stroke.

[5] Inouye SK, Westendorp RGJ, Saczynski JS (2014). Delirium in elderly people. Lancet Lond Engl.

[6] McCusker J, Cole M, Abrahamowicz M, Primeau F, Belzile E (2002). Delirium predicts 12-month mortality. Arch Intern Med.

[7] Rollo E, Callea A, Brunetti V, Vollono C, Marotta J, Imperatori C (2021). Delirium in acute stroke: a prospective, cross-sectional, cohort study. Eur J Neurol..

[8] Ouimet S, Kavanagh BP, Gottfried SB, Skrobik Y (2007). Incidence, risk factors and consequences of ICU delirium. Intensive Care Med.

[9] Cunningham C, MacLullich AM (2013). At the extreme end of the psychoneuroimmunological spectrum: delirium as a maladaptive sickness behaviour response. Brain Behav Immun.

[10] Hshieh TT, Fong TG, Marcantonio ER, Inouye SK (2008). Cholinergic deficiency hypothesis in delirium: a synthesis of current evidence. J Gerontol A Biol Sci Med Sci.

[11] Guillamondegui OD, Richards JE, Ely EW, Jackson JC, Archer KR, Archer-Swygert K (2011). Does hypoxia affect intensive care unit delirium or long-term cognitive impairment after multiple trauma without intracranial hemorrhage?. J Trauma.

[12] Ernst G, Watne LO, Rostrup M, Neerland BE (2020). Delirium in patients with hip fracture is associated with increased heart rate variability. Aging Clin Exp Res.

[13] Jooyoung Oh null, Dongrae Cho null, Jongin Kim null, Jaeseok Heo null, Jaesub Park null, Se Hee Na null et al. Changes in heart rate variability of patients with delirium in intensive care unit. Annu Int Conf IEEE Eng Med Biol Soc IEEE Eng Med Biol Soc Annu Int Conf. 2017;2017:3118–21 10.1109/EMBC.2017.8037517 29060558

[14] Karol DE, Muzyk AJ, Preud’homme XA (2011). A case of delirium, motor disturbances, and autonomic dysfunction due to baclofen and tizanidine withdrawal: a review of the literature. Gen Hosp Psychiatry.

[15] Neerland BE, Wyller TB, Wyller VBB (2019). Autonomic cardiovascular control in older patients with acute infection and delirium: a pilot study of orthostatic stress responses. BMC Geriatr.

[16] Oh J, Cho D, Park J, Na SH, Kim J, Heo J (2018). Prediction and early detection of delirium in the intensive care unit by using heart rate variability and machine learning. Physiol Meas..

[17] Zaal IJ, van der Kooi AW, van Schelven LJ, Oey PL, Slooter AJC (2015). Heart rate variability in intensive care unit patients with delirium. J Neuropsychiatry Clin Neurosci.

[18] van Swieten JC, Koudstaal PJ, Visser MC, Schouten HJ, van Gijn J (1988). Interobserver agreement for the assessment of handicap in stroke patients. Stroke.

[19] Ely EW, Margolin R, Francis J, May L, Truman B, Dittus R (2001). Evaluation of delirium in critically ill patients: validation of the confusion assessment method for the intensive care unit (CAM-ICU). Crit Care Med.

[20] Sessler CN, Gosnell MS, Grap MJ, Brophy GM, O’Neal PV, Keane KA (2002). The Richmond agitation-sedation scale: validity and reliability in adult intensive care unit patients. Am J Respir Crit Care Med.

[21] Niskanen J-P, Tarvainen MP, Ranta-Aho PO, Karjalainen PA (2004). Software for advanced HRV analysis. Comput Methods Programs Biomed.

[22] Champéroux P, Fesler P, Judé S, Richard S, Le Guennec J-Y, Thireau J (2018). High-frequency autonomic modulation: a new model for analysis of autonomic cardiac control. Br J Pharmacol.

[23] (1996) Heart rate variability. Eur Heart J 17:28 10.1093/oxfordjournals.eurheartj.a014827 8732364

[24] Faul F, Erdfelder E, Buchner A, Lang A-G (2009). Statistical power analyses using G*Power 3.1: tests for correlation and regression analyses. Behav Res Methods.

[25] Faul F, Erdfelder E, Lang A-G, Buchner A (2007). G*Power 3: a flexible statistical power analysis program for the social, behavioral, and biomedical sciences. Behav Res Methods.

[26] Tan C, Saito N, Miyawaki I (2017). Changes in heart rate and autonomic nervous activity after orthopedic surgery in elderly Japanese patients. Kobe J Med Sci.

[27] van den Berg MP, Haaksma J, Brouwer J, Tieleman RG, Mulder G, Crijns HJ (1997). Heart rate variability in patients with atrial fibrillation is related to vagal tone. Circulation.

[28] Nakamizo T, Kanda T, Kudo Y, Sugawara E, Hashimoto E, Okazaki A (2020). Development of a clinical score, PANDA, to predict delirium in stroke care unit. J Neurol Sci..

[29] Oldenbeuving AW, de Kort PLM, Jansen BPW, Algra A, Kappelle LJ, Roks G (2011). Delirium in the acute phase after stroke: incidence, risk factors, and outcome. Neurology.

[30] Shaw R, Drozdowska B, Taylor-Rowan M, Elliott E, Cuthbertson G, Stott DJ (2019). Delirium in an acute stroke setting, occurrence, and risk factors. Stroke.

[31] Kotfis K, Bott-Olejnik M, Szylińska A, Listewnik M, Rotter I (2019). Characteristics, risk factors and outcome of early-onset delirium in elderly patients with first ever acute ischemic stroke - a prospective observational cohort study. Clin Interv Aging.

[32] Qu J, Chen Y, Luo G, Zhong H, Xiao W, Yin H (2018). Delirium in the acute phase of ischemic stroke: incidence, risk factors, and effects on functional outcome. J Stroke Cerebrovasc Dis Off J Natl Stroke Assoc.

[33] Maclullich AMJ, Ferguson KJ, Miller T, de Rooij SEJA, Cunningham C (2008). Unravelling the pathophysiology of delirium: a focus on the role of aberrant stress responses. J Psychosom Res.

[34] Papathanassoglou EDE, Skrobik Y, Hegadoren K, Thompson P, Stelfox HT, Norris C (2019). Relaxation for critically ill patient outcomes and stress-coping enhancement (REPOSE): a protocol for a pilot randomised trial of an integrative intervention to improve critically ill patients’ delirium and related outcomes. BMJ Open..

[35] Vilke GM, DeBard ML, Chan TC, Ho JD, Dawes DM, Hall C (2012). Excited delirium syndrome (ExDS): defining based on a review of the literature. J Emerg Med.

[36] Kleiger RE, Miller JP, Bigger JT, Moss AJ (1987). Decreased heart rate variability and its association with increased mortality after acute myocardial infarction. Am J Cardiol.

[37] La Rovere MT, Bigger JT, Marcus FI, Mortara A, Schwartz PJ (1998). Baroreflex sensitivity and heart-rate variability in prediction of total cardiac mortality after myocardial infarction. ATRAMI (Autonomic Tone and Reflexes After Myocardial Infarction) Investigators. Lancet Lond Engl.

[38] Wijbenga JA, Balk AH, Meij SH, Simoons ML, Malik M (1998). Heart rate variability index in congestive heart failure: relation to clinical variables and prognosis. Eur Heart J.

[39] Binder T, Frey B, Porenta G, Heinz G, Wutte M, Kreiner G (1992). Prognostic value of heart rate variability in patients awaiting cardiac transplantation. Pacing Clin Electrophysiol PACE.

[40] Lees T, Shad-Kaneez F, Simpson AM, Nassif NT, Lin Y, Lal S (2018) Heart rate variability as a biomarker for predicting stroke, post-stroke complications and functionality. Biomark Insights. 18(13):1177271918786931 10.1177/1177271918786931 PMC6052496 30038486

[41] Cripps TR, Malik M, Farrell TG, Camm AJ (1991). Prognostic value of reduced heart rate variability after myocardial infarction: clinical evaluation of a new analysis method. Br Heart J.

[42] Fei L, Copie X, Malik M, Camm AJ (1996). Short- and long-term assessment of heart rate variability for risk stratification after acute myocardial infarction. Am J Cardiol.

[43] Stein PK, Domitrovich PP, Hui N, Rautaharju P, Gottdiener J (2005). Sometimes higher heart rate variability is not better heart rate variability: results of graphical and nonlinear analyses. J Cardiovasc Electrophysiol.

[44] Pasińska P, Wilk A, Kowalska K, Szyper-Maciejowska A, Klimkowicz-Mrowiec A (2019). The long-term prognosis of patients with delirium in the acute phase of stroke: PRospective Observational POLIsh Study (PROPOLIS). J Neurol.

[45] Inouye SK, Bogardus ST, Charpentier PA, Leo-Summers L, Acampora D, Holford TR (1999). A multicomponent intervention to prevent delirium in hospitalized older patients. N Engl J Med.

